# Effects of Illitic Clay on the Phases, Microstructure, Physical Properties and Pyroplastic Deformation of Industrial Slag Ceramics

**DOI:** 10.3390/ma16010233

**Published:** 2022-12-27

**Authors:** Hao You, Hongjuan Sun, Tongjiang Peng, Xin Zhou, Li Chao, Can Wang

**Affiliations:** 1 Key Laboratory of Ministry of Education for Solid Waste Treatment and Resource Recycle, Southwest University of Science and Technology, Mianyang 621010, China; 2 Institute of Mineral Materials and Applications, Southwest University of Science and Technology, Mianyang 621010, China; 3 Sichuan Engineering Laboratory of Nonmetallic Mineral Powder Modification & High-Quality Utilization, Southwest University of Science and Technology, Mianyang 621010, China; 4 Center of Forecasting and Analysis, Southwest University of Science and Technology, Mianyang 621010, China

**Keywords:** phases, microstructure, physical properties, pyroplastic deformation, industrial slag ceramics

## Abstract

Preparing ceramic materials is a meaningful way to treat and utilize industrial slags. In this work, high-performance and low-deformation industrial slag ceramics were prepared from Ti-extraction blast furnace slag and illitic clay. The phase composition and contents, microstructure, physical properties, and pyroplastic deformation of ceramic samples were investigated. With the increasing proportion of illitic clay, the main crystalline phase of ceramic samples changed from akermanite to Fe-bearing diopside. Moreover, the minor crystalline phases changed from perovskite and spinel to anorthite and titanite. The proportion of illitic clay was linearly related to the amorphous phase content. The dense microstructure comprised concentrated short-columnar and granular grains with a few isolated pores, whereas plate-like grains destroyed their denseness. An appropriate proportion of illitic clay helped to improve the physical properties, increase the high-temperature viscosity and reduce the deformation of the ceramics. The optimal proportion of illitic clay was 30%, and the prepared ceramic sample had a dense microstructure and excellent physical properties. Its bulk density was 2.82 g/cm^3^, bending strength was 62.17 MPa, and water absorption was 0.21%.

## 1. Introduction

Industrial slag is a by-product formed when generating industrial products. Due to the different production methods and original sources, the properties of industrial slag are complex and diverse. The addition of environmentally-hazardous gases and liquids in the production process of industrial slag cause the problems associated with corrosion, toxicity, and pollution. Therefore, the resource utilization of industrial slag is an important challenge in the field of solid waste management [1,2,3]. Preparing ceramic materials from industrial slags has been acknowledged as an effective way of solid waste treatment and utilization. Traditional ceramics are mainly made up of natural minerals, including clays, feldspar, and quartz sand. Industrial slag ceramics are novel ceramics prepared from industrial slags by replacing all or parts of natural minerals. Compared with the traditional K_2_O (Na_2_O)-SiO_2_-Al_2_O_3_ ternary system ceramics, industrial slag ceramics have the following advantages: (1) More abundant ceramic systems: such as CaO-MgO-Al_2_O_3_-SiO_2_, CaO-MgO-Al_2_O_3_-SiO_2_-Fe_2_O_3_, and SiO_2_-MgO-CaO-Fe_2_O_3_. (2) More flexible ceramic components: the CaO, MgO, and Fe_2_O_3_ contents of industrial slag ceramics can be above 10%, while they must be less than 2% in traditional ceramics. (3) More solid waste utilization: one or more industrial slags are utilized as raw materials, and their proportion in the formula can be up to 100%. (4) More specialized functions: adjusting the pore structure to make the ceramic acquire sound absorption and thermal insulation, generating unique crystalline phases for degrading organic pollutants [4,5,6,7,8]. Nevertheless, the development and preparation of industrial slag ceramics still present some challenges. The complex composition of the slag may limit the firing system. For example, when the CaO content in the slag is high, its participation in the preparation of ceramic tiles suffers from a narrow range of sintering temperatures and easy deformation at high temperatures [4,9]. While the high MgO content may excessively increase the sintering temperature of ceramic tiles (>1200 °C), causing a thermal energy transition consumption and an increase in preparation costs [10]. Some volatile components at high temperatures may leave bubbles or pinholes on the surface and inside ceramic materials, affecting their performance and aesthetics. Industrial solid wastes, such as steel slag, blast furnace slag, fly ash, and tailings, have been used as raw materials to prepare stoneware tiles, floor tiles, wall tiles, and porous tiles [11,12,13,14]. However, most of the research on industrial slag ceramics is done in their laboratory preparation stage and rarely enters into actual production. No matter what, the development and production of industrial slag ceramics will be the future trend of ceramic materials.

Ti-extraction blast furnace slag (EBFS) is an industrial slag generated from Ti-bearing blast furnace slag after the “high-temperature carbonization and low-temperature chlorination” extract titanium process in the Panxi region of China [8,15]. Studies on EBFS are mainly related to the preparation of materials (foamed concrete, ceramics, cementitious materials, etc.), dechlorination, and recovery of valuable components [15,16,17,18,19,20]. Xi et al. prepared porous glass-ceramics with EBFS and waste glass, and the effects of EBFS amount, heat treatment system, and flux amount on the properties of porous glass-ceramics were investigated in detail [7,21]. Hui et al. prepared self-glazed foam ceramics with EBFS and gold tailings, and the evolution of porosity and properties were studied thoroughly [22]. Our previous studies proved that EBFS was applied to prepare dense glass-ceramics and functional ceramics [8,23]. The phases, microstructure, and physical properties of ceramic materials prepared from EBFS have been initially recognized, but the deformation of these ceramic materials has not received sufficient attention.

Illite is a potassium-rich dioctahedral silicate mica-like clay mineral with T-O-T type and forms a series with interlayer cation content from 0.6 to 0.85. Illitic clay always contains illite, as well as montmorillonite, muscovite, kaolinite, quartz, and other minerals, and it is an important natural source for ceramics [24,25,26]. Aras investigated the phase transformation of ceramic bodies with different clay compositions at different firing temperatures and showed that cristobalite and mullite do not readily form in illite/sericite-rich ceramic bodies [27]. Ferrari and Gualtieri discussed the effect of illite content in illitic clay on the phase composition and physical properties of ceramic tiles. They found that the higher the illite content of the clay, the greater the amorphous phase content, and the lower the mullite and quartz content [24]. Knapek et al. investigated the microcrack growth inside illite-based ceramics during cooling and its effect on ceramic properties. The results showed that microcracks inside the ceramics were produced by the mismatch between the thermal expansion coefficients of glass and quartz [28]. Magagnin et al. found that illite effectively reduced the pyroplastic deformation of porcelain stoneware tiles [29]. Thus, illitic clay is an excellent raw material for adjusting the phases of ceramic materials, enhancing their physical properties, and resisting their deformation.

Pyroplastic deformation is one of the main defects of ceramic materials. Pyroplastic deformation is the bending of ceramics caused by gravity during heat treatment and can be defined as the loss of shape of ceramic tiles during firing, and it is also an important indicator to evaluate the firing stability of ceramics tiles [30,31]. The pyroplastic deformation is quantified by the pyroplastic deformation index (PI), representing the deformation tendency of a specimen with fixed dimensions subjected to gravity during firing. As PI increase, the deformation of the fired specimen also increases. Research on the pyroplastic deformation of traditional ceramics, such as whitewares, porcelain stonewares, and vitreous ceramics, has been conducted sufficiently. However, that is still in the initial stage for industrial slag ceramics [32,33,34].

In this work, industrial slag ceramics were prepared from EBFS and illitic clay (IC). Firstly, the phases and chemical compositions of raw materials were investigated. Secondly, the phase compositions and contents, cell parameters of Fe-bearing diopside and microstructure of ceramic samples with different proportion of IC were characterized and calculated. Several physical properties, PI and viscosity of ceramics (E_p_) of these samples were measured. Finally, the optimal proportion of IC was determined by physical properties and PI values. Dense, high-performance, and low-deformation industrial slag ceramics were obtained. This work demonstrates how illitic clay affects industrial slag ceramics’ phases, structure, physical properties, and pyroplastic deformation. Further, it provides experimental support for developing and preparing industrial slag ceramics.

## 2. Materials and Methods

### 2.1. Raw Materials

EBFS and IC were collected from Panzhihua, China. To avoid the oxidation of residual carbon and the volatilization of chlorides during the rapid heating process leading to the cracking of the green bodies of ceramics, EBFS was roasted at 900 °C for 1 h to remove residual carbon and chlorides as much as possible and to obtain roasted Ti-extraction blast furnace slag (REBFS). REBFS and IC were used as raw materials to prepare industrial slag ceramics. The loss on ignition (LOI) of the three raw materials was measured according to GB/T176-2008 standard. The obtained LOI was used to adjust the chemical composition of each raw material, and the results are shown in Table 1.

### 2.2. Preparation of Ceramics

The REBFS and IC were weighed according to the formulas and proportions in Table 2. Subsequently, they were wet ground for 1 h in a ball mill, and the slurry was passed through an 80 mesh sieve and dried at 105 ± 5 °C. The dried finely ground powder was added with 8% mass of polyvinyl alcohol solution (5% m/v) and mixed homogeneously. After filtering with a 20 mesh screen, the mixture was pelletized and then stored for 1 day. The pelletized particles were pressed into Ø 25.5 mm × 5.5 mm cylindrical disks and 50 mm × 5 mm × 5 mm rectangular bars as green bodies. The green bodies were placed in a muffle furnace, and the initial temperature was increased to 1170 °C at a heating rate of 10 °C/min and held for 30 min. After cooling, ceramic samples were obtained and named IC-00, IC-10, IC-20, IC-30, IC-40, and IC-50, respectively, according to the proportion of IC.

### 2.3. Characterization and Testing

#### 2.3.1. Sample Characterization

The chemical compositions of raw materials were identified by X-ray fluorescence spectroscopy (XRF, Axios, PANalytical, Almelo, The Netherlands). The phase analysis of raw materials and ceramic samples was examined by X-ray diffraction (XRD, Ultima IV, Rigaku, Japan) using Cu Kα radiation at operating conditions of 40 kV voltage, 40 mA current, 2θ range from 3–80°, and a scanning speed of 4°(2θ)min^−1^ for ceramics and 15°(2θ)min^−1^ for raw materials. The “Whole Pattern Fit” module in Jade was used for quantitative analysis and structural refinement of phases. The background curve is Fixed Background, the profile shape function is pseudo-Voigt, the RIR value of the amorphous part is set to 10.00, and the refinement range is 2θ = 10–80°. The refinement process is referred to works of Zhao and Tan [35]. R-factor value and E-factor value are considered as the criteria for judging the quality of refinement. The ratio of R/E is called the “goodness of fit”, which is approximately 1, the refinement results are better [36]. The particle size distribution of raw materials was determined by a laser particle size analyzer (PSA, LS13320, Beckman, Pasadena, CA, USA). The microstructures of ceramic samples were observed by scanning electron microscopy (SEM, Sigma300, Carl Zeiss, Germany). SEM photos were used to analyze and calculate the pore sizes and their distribution by Image-Pro Plus.

#### 2.3.2. Physical Properties Testing

The bulk density and water absorption of ceramic samples were measured by the Archimedes method using an electronic analytic balance (WT5003GHS, Changzhou Wantai Balance Instrument Co., LTD., Changzhou, China). The linear shrinkage of the samples was calculated according to Equation (1). The apparent porosity of the samples was calculated according to Equation (2) [14]. The rupture load value of the samples was tested by using an electronic universal testing machine (ETM305F-2, WANCE, China). The loading speed was kept at 0.5 mm/min and span at 35 mm. The bending strength of the samples was measured by the three-point bending method and the equation was (3) [37].

(1) $$L_{s}=\frac{L_{1}-L_{2}}{L_{1}}\times 100\%$$

(2) $$P_{o}=\frac{M_{f}-M_{d}}{M_{f}-M_{s}}$$

(3) $$\sigma =\frac{3FL_{b}}{2bt^{2}}$$

where *L_s_* is the linear shrinkage (%), *L*_1_ is the diameter of cylindrical disk (mm), *L*_2_ is the diameter of the sample (mm), *P_o_* is the apparent porosity (%), *M_d_* is the mass of the dry sample (g), *M_s_* is the mass of the sample immersed in water (g), *M_f_* is the mass of the wet sample after water absorption (g), σ is the bending strength (MPa), *F* is the rupture load value (N), *L_b_* is the distance between the supports (mm), *b* is the width of sample (mm), and *t* is its thickness (mm).

#### 2.3.3. Pyroplastic Deformation Testing

**Figure 1:**
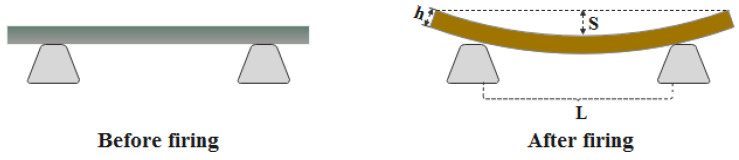
Position of the green bar and deformed sample before and after firing.

In order to test and analyze the pyroplastic deformation of ceramic samples, 50 mm × 5 mm × 5 mm rectangular bars were placed on the trapezoidal supports. These bars were fired following the heat treatment schedule in Section 2.2. The process of pyroplastic deformation of ceramics is shown in Figure 1. Some necessary parameters of the deformed samples were collected and measured. After that, the PI and *E_p_* were calculated according to Equations (4) and (5), respectively [30,38,39].

(4) $$\mathrm{PI}=\frac{4Sh^{2}}{3L^{4}}$$

(5) $$E_{p}=\frac{5g\rho_{b}L^{4}}{32Sh^{2}}$$

where PI is the pyroplastic deformation index (cm^−1^), *S* is the maximum deformation (mm), *g* is the gravitational constant, *ρ_b_* is the bulk density of the fired sample (g/cm^3^), *L* is the distance between the trapezoidal supports (mm), *E_p_* is the viscosity (Pa × s), and *h* is the sample thickness (mm).

## 3. Results and Discussion

### 3.1. Attributes and Functions of Raw Materials

The Cl content of EBFS was as high as 8.96%, whereas that of REBFS decreased to 2.70% (Table 1). 69.87% Cl was removed by the roasting process, which confirms that roasting effectively removes chlorine from EBFS. REBFS had 31.63% CaO, which could be used as a CaO source for industrial slag ceramics and participate in the formation of Ca-containing crystalline phases. IC had 48.13% SiO_2_ and 15.84% Al_2_O_3_, and these components play significant roles in the widening of the firing temperature, improving the firing stability and reducing deformation of ceramics. R_2_O as a fluxing component accelerates the rate of sintering densification. TiO_2_ and Fe_2_O_3_ act as nucleating agents to improve crystallinity and promote the growth of crystalline phases [40].

**Figure 2:**
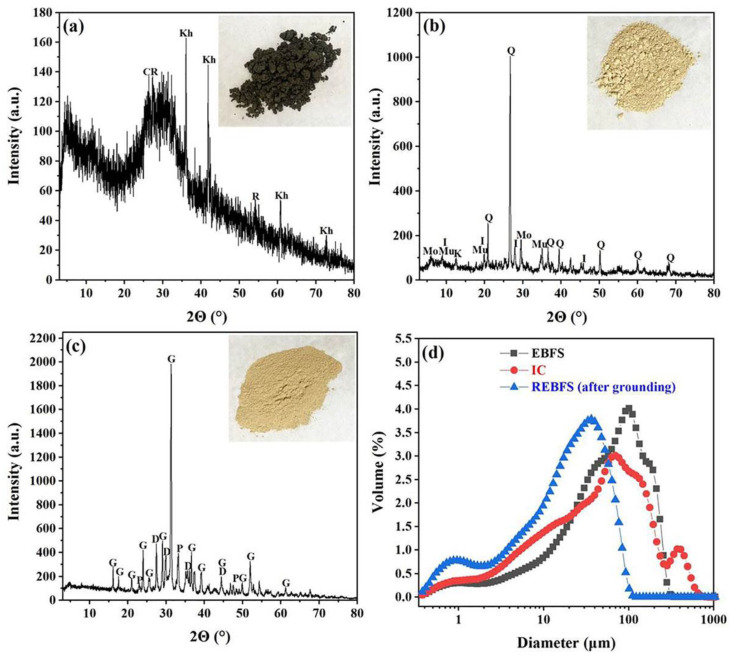
Phase compositions and particle properties of raw materials. (**a**–**c**) XRD patterns of EBFS, IC, and REBFS, respectively. (**d**) Particle size distribution. C = Carbon, Kh = Khamrabaevite, R = Rutile, G = Gehlenite, D = Diopside, P = Perovskite, Q = Quartz, I = Illite, Mo = Montmorillonite, Mu = Muscovite, K = Kaolinite.

Figure 2 shows the phase compositions and particle size distribution of EBFS, IC, and REBFS. From Figure 2a, EBFS had a large number of amorphous phases, and the crystalline phases were khamrabaevite (PDF#73-0472), rutile (PDF#75-1478), and carbon (PDF#75-1621). After roasting, almost all amorphous phases were converted to crystalline, resulting in gehlenite (PDF#72-2128), diopside (PDF#75-1092), and perovskite (PDF#82-0228) forming in REBFS (Figure 2c). IC was composed of quartz (PDF#79-1906), illite (PDF#26-0911), muscovite (PDF#84-1302) and trace of kaolinite (PDF#78-2110) and montmorillonite (PDF#13-0135) (Figure 2b). It had a low illite content and high impurity minerals. The particle size of EBFS was mainly concentrated in 10-180 μm with d (0.1), d (0.5), and d (0.9) of 7.09 μm, 61.73 μm, and 181.42 μm, respectively. IC had a broad particle size distribution, and its d (0.1), d (0.5), and d (0.9) were 4.36 μm, 48.73 μm, and 205.60 μm, respectively. The wet-ground REBFS showed a narrow size distribution and finer particles, mainly in the range of 1–60 μm, while d (0.1), d (0.5), and d (0.9) were 1.81 μm, 20.40 μm, and 59.24 μm, respectively.

### 3.2. Phase Composition and Relative Contents of the Ceramic Samples

**Figure 3:**
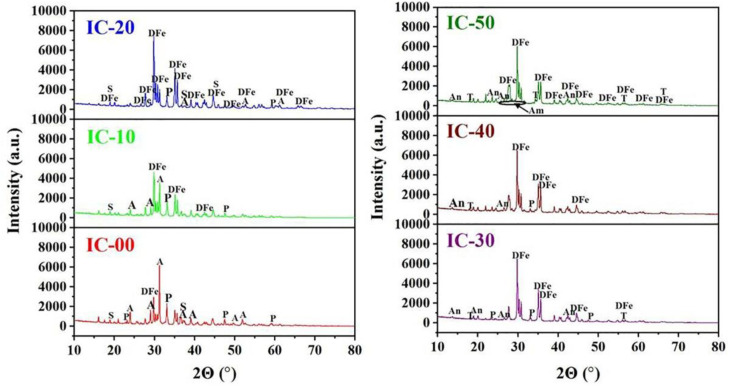
XRD patterns of the ceramic samples. A = Akermanite, DFe = Fe-bearing diopside, P = Perovskite, S = Spinel, An = Anorthite, T = Titanite, Am = Amorphous.

Figure 3 shows the XRD patterns of the fired samples with different proportions of IC. From IC-00 to IC-20, the crystalline phases were akermanite (ICSD#20391), Fe-bearing diopside (ICSD#83821), perovskite (ICSD#74212), and spinel (ICSD#31377). With increasing the proportion of IC (0–20%), the intensity of the diffraction peaks of akermanite and spinel decreased, but that of Fe-bearing diopside increased. It indicates that Fe-bearing content increased, while those of akermanite and spinel decreased. The decrease in CaO/SiO_2_ ratio in the ceramic components made it easier to develop diopside than akermanite in the ceramic samples. A lower percentage of MgO might affect the synthesis of spinel. Ionic substitution occurred regularly in diopside, Fe^2+^ substituted Mg^2+^, and Fe^3+^ substituted Al^3+^/Si^4+^ in cells that participate in diopside synthesis, therefore producing diopside with higher Fe content [5,41,42]. From IC-30 to IC-50, Fe-bearing diopside continuously existed in ceramics, but spinel and akermanite were eliminated, while anorthite (ICSD#86330) and titanite (ICSD#24614) were further detected. With increasing the proportion of IC (30–50%), the intensity of diffraction peaks of Fe-bearing diopside decreased, but that of anorthite increased gradually. The decrease in the MgO/Al_2_O_3_ ratio and the non-equivalent electric charge substitution from Al^3+^ to Mg^2+^ in the octahedral position and Si^4+^ in the tetrahedral position favored the growth of anorthite instead of Fe-bearing diopside [43,44,45]. Except for the IC-50 sample, other samples contained perovskite, and the intensity of diffraction peaks decreased until they disappeared. The decrease of TiO_2_ in the ceramic components might be the origin of this effect. As for the perovskite-type oxides, Si^4+^ can substitute and occupy the B-site ion in the crystal lattice. Moreover, the partial replacement of Ti^4+^ by Si^4+^ drove the conversion from CaTiO_3_ to CaTiSiO_5_. These explained the decrease of CaTiO_3_ content but the increase of CaTiSiO_5_ content [46].

The species of crystalline phases in the raw material and ceramic samples were significantly different. Mechanical ball milling distorted the crystalline layer lattice of 2:1 and 1:1 type minerals, making these types of minerals in the illitic clay easy to decompose. When further treated by high temperatures, clay minerals’ lattice structure was destroyed and converted into amorphous liquid. As a result, clay minerals were not found in the ceramic samples [47]. Quartz was not detected in the XRD patterns of the ceramics, which may be attributable to the participation in the synthesis and reaction of the ceramic phases.

An apparent “bulge” was observed at 2θ = 26–33° in the XRD pattern of the IC-50 sample, indicating a certain amount of amorphous phase existed. The XRD patterns provided in Figure 3 were refined and quantitatively calculated using the Rietveld method to measure the contents of phases and analyze their variations accurately. Table 3 shows the refinement factors and R/E. The R-factors and E-factors were both low, and the R/E values were close to 1 and less than 1.5, demonstrating a solid fit and a good refinement.

**Figure 4:**
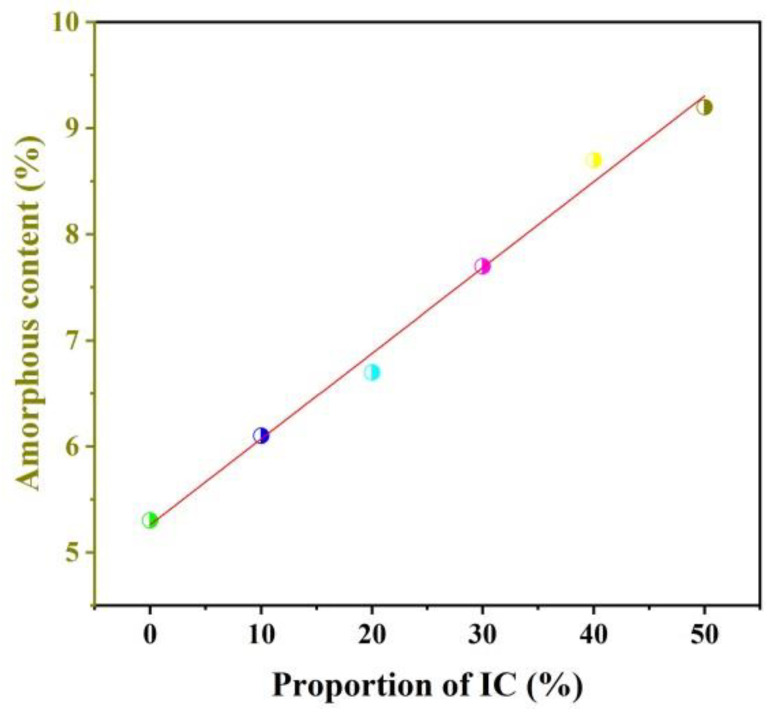
Proportion of IC vs. amorphous content.

Table 4 illustrates the relative contents of each phase. The contents of akermanite and spinel were dramatically decreased and that of Fe-bearing diopside was significantly increased from IC-00 to IC-20. Afterward, the content of akermanite and spinel decreased to 0% and that of Fe-bearing diopside reached a maximum of 78.3%, while 9.1% anorthite and 0.5% titanite were present in the IC-30 sample. However, when the proportion of IC was above 30%, the content of Fe-bearing diopside decreased approximately 27.8%, and anorthite increased approximately 26.5%, suggesting that parts of Fe-bearing diopside converted to anorthite. The content of perovskite decreased approximately 4.4% and that of titanite increased 4.2%, demonstrating that titanite should be converted from perovskite. It was worth noting that the content of the amorphous phase increased with the proportion of IC rose. Their relationship was linear and as follows: y = 0.0809x + 5.2619, R^2^ = 0.9906 (Figure 4), which was consistent with the finding of Ferrari and Gualtieri [24].

### 3.3. Structural Distortion of Fe-Bearing Diopside

The degree of structural distortion of crystals is typically reflected by the evolution of cell parameters and volumes. These variations also reflect the growing tendency of the crystal. From Table 5, with the addition of IC, the volume of a single cell of Fe-bearing diopside increased from 440.6 Å^3^ to 443.4 Å^3^. The length of the a-axis continued to increase, the length of the b-axis decreased and then increased, the variation of the c-axis length was opposite to the b-axis, and the β-angle fluctuated slightly. These changes showed that the addition of IC promoted the growth of Fe-bearing diopside cells while the crystal structure was slightly distorted. The structural distortion should be mainly related to ionic substitution occurring in Fe-bearing diopside. Crystal growth and structural distortion led to alterations of grain morphology, which may change the extension of microcracks and the state of pores in ceramics. These features certainly influence the stability and denseness of the ceramic structure [48,49].

### 3.4. Microscopic Structure of the Ceramic Samples

**Figure 5:**
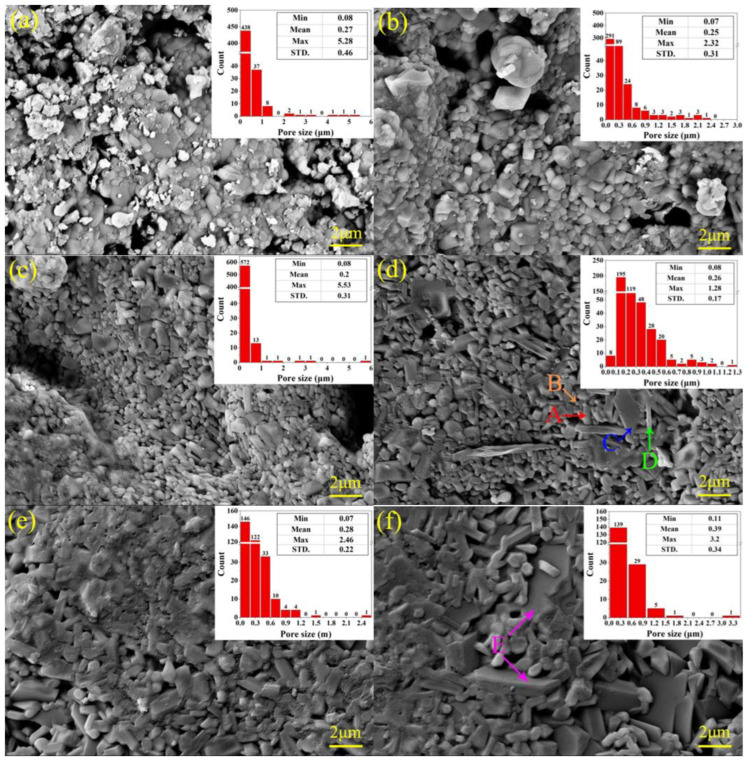
SEM photos of the ceramic samples. (**a**) IC-00 sample, (**b**) IC-10 sample, (**c**) IC-20 sample, (**d**) IC-30 sample, (**e**) IC-40 sample, and (**f**) IC-50 sample. A: short-columnar, B: granular, C: flake, D: needle-like, and E: plate-like.

Figure 5 shows the grain morphology, the pore sizes and their distribution in the ceramic samples. The microstructures of the IC-00, IC-10 and IC-20 samples were loose, and they both had many apparent pores with different sizes. The number of pores with a size larger than 1 μm and the mean value of all pore sizes decreased with increasing the proportion of illitic clay, which indicates that the addition of illitic clay can effectively reduce the porosity of ceramics and improve their denseness. From Figure 5d, the state and distribution of pores turned from interconnected to isolated, and almost all pore sizes were lower than 1 μm. The maximum pore size was only 1.28 μm, suggesting that the IC-30 sample had a relatively dense microstructure. The surface energy created by these tiny pores pushes the viscous flow of the liquid phase and causes it to fill the gaps between grains, thus increasing the density of the ceramic [50]. The unique morphology of the grains was able to be observed in Figure 5d. Short columnar shapes are the dominant morphology of these grains, followed by granular shapes and minor flakes and needles. According to the results of XRD analysis and phase content calculations, short columnar grains represented Fe-bearing diopside, and flakes and needles belonged to anorthite. Most granular grains were probably Fe-bearing diopside, with the rest being perovskite and titanite [51,52]. Compared with the IC-30 sample, the mean and maximum values of pore sizes in the IC-40 and IC-50 samples increased from 0.26 μm and 1.28 μm to 0.39 μm and 3.2 μm, respectively. This implies that excessive illitic clay may increase the amount and size of apparent pores in the ceramics, which is not conducive to structural denseness. The addition of illitic clay promoted the growth of Fe-bearing diopside grains (Table 5), resulting in larger voids among the interlaced grains. Plate-like grains appeared in the IC-50 sample, developed from flaky and needle-like anorthite (Figure 5f). These plate-like grains destroyed the tight aggregation of grains and formed apparent secondary pores among the grains. Therefore, a suitable proportion of illite clay (30%) is beneficial to improve the denseness and reduce the porosity of the ceramics, and an excessive proportion is detrimental.

### 3.5. Physical Properties of the Ceramic Samples

**Figure 6:**
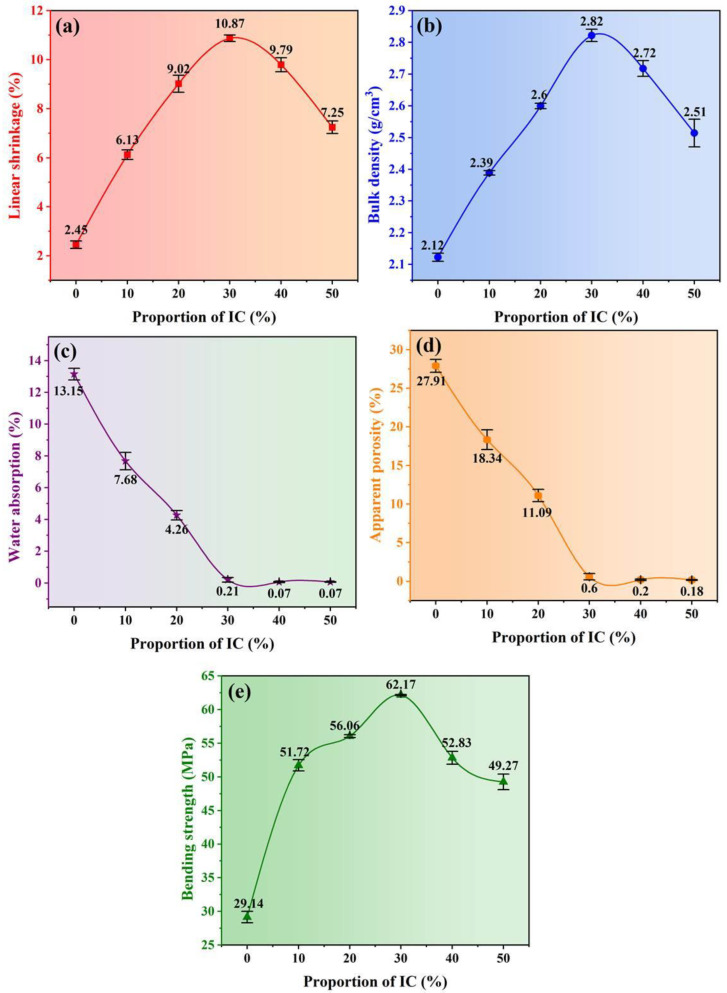
Physical properties of the ceramic samples. (**a**) linear shrinkage, (**b**) bulk density, (**c**) water absorption, (**d**) apparent porosity, and (**e**) bending strength.

Figure 6 displays the several physical properties of the ceramic samples. In general, the linear shrinkage, bulk density and bending strength of the samples firstly increased and then decreased with increasing the proportion of IC (Figure 6a,b,e). The appropriate proportion of IC contributes to improving the densification and mechanical strength of ceramics, which corresponds to the SEM analysis results. We consider that the main reasons for these changes were twofold. On the one hand, Fe-bearing diopside had a significant influence on the mechanical properties. The variation tendency of Fe-bearing diopside content was consistent with that of bending strength. This positive correlation implied that increasing the Fe-bearing diopside content was beneficial to improve the bending strength of the ceramic samples. On the other hand, the morphology and aggregation of grains played an important role in determining the denseness of ceramics. Granular and short columnar grains aggregated tightly among them dense the microstructure. In contrast, plate-like grains destroyed this aggregation and led to a loose way. Therefore, increasing Fe-bearing diopside content and controlling the morphology of the crystalline grains are the critical factors to improve the denseness and mechanical strength of industrial slag ceramics. The water absorption and apparent porosity decreased with first occurring quickly and then slowing down (Figure 6c,d). It is validated information that the particle size, the heat treatment, the flux, phase content, and the microstructure could influence the variation of water absorption and porosity of ceramics [25,53,54,55,56]. In this study, we kept the same particle size and heat treatment condition, and no addition flux for all samples. It is worth analyzing how the microstructure and phase content affect the water absorption and the apparent porosity of the ceramics. The mutual aggregation of short columnar and granular grains changed the pore state (interconnected to isolated) and pore size, and the number of pores larger than 1 μm and the maximum pore size decreased (Figure 5a–d), resulting in a rapid drop in the water absorption and apparent porosity of the IC-00 to IC-30 samples. The partial disruption of the dense structure and the formation of secondary pores were attributed to the appearance and growth of plate-like grains. However, the continued increase in the amorphous phase content (Table 4) might have slowed down this structural disruption, allowing a slight increase in the mean and maximum values of pores but a decrease in the statistical number of pores. As a result, the water absorption and apparent porosity of the IC-40 and IC-50 samples were at deficient levels.

According to the above variations of physical properties, it can be assumed that the crystalline phase content and grain morphology are more influential on the denseness and mechanical strength of the samples, while the amorphous phase content and the aggregation of grains are the key factors to alter the water absorption and apparent porosity of the samples. IC-30 samples had the optimal physical properties, with average values of linear shrinkage, bulk density, and bending strength of 10.87%, 2.82 g/cm^3^, and 62.17 MPa, respectively, and average values of water absorption and apparent porosity of 0.21% and 0.60%, respectively. The above-mentioned physical properties highly support the phase and microstructure obtained from the XRD and SEM results. At the same time, the optimal proportion of IC was 30%, was further determined.

### 3.6. Pyroplastic Deformation of the Ceramic Samples

**Figure 7:**
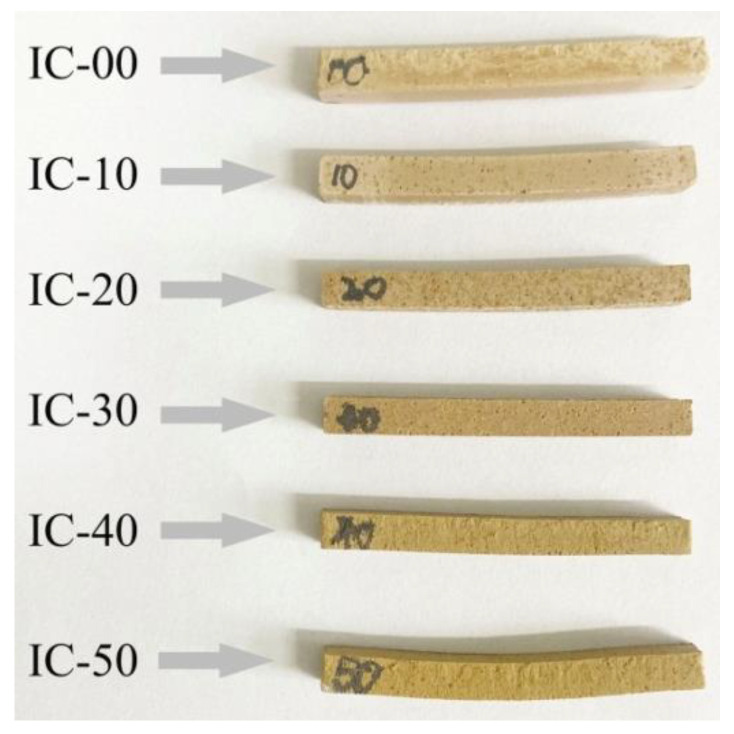
Photos of pyroplastic deformation state of the ceramic samples.

Figure 7 shows the pyroplasitc deformation state of the IC-00 to IC-50 samples. With increasing the proportion of IC, the deformation of the samples followed firstly reducing and then expanding. The IC-30 sample had the minimum deformation and no obvious warping, while the IC-50 sample had the maximum deformation and severe warping.

**Figure 8:**
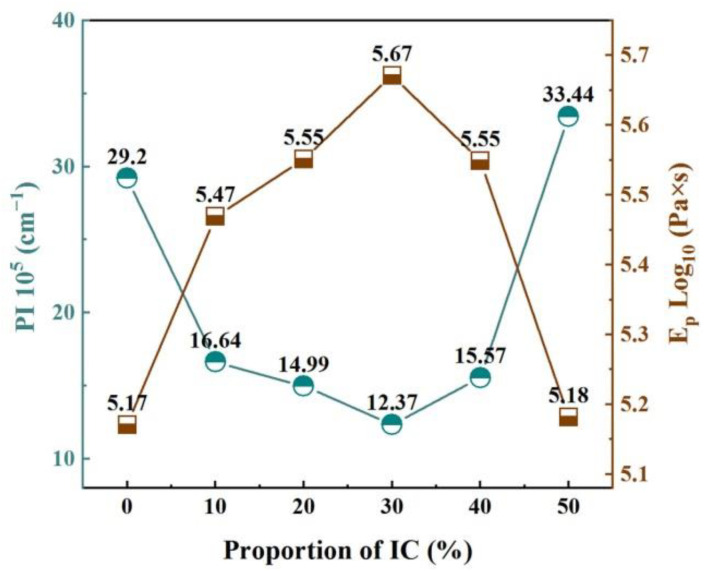
PI and E_p_ of the ceramic samples.

**Figure 9:**
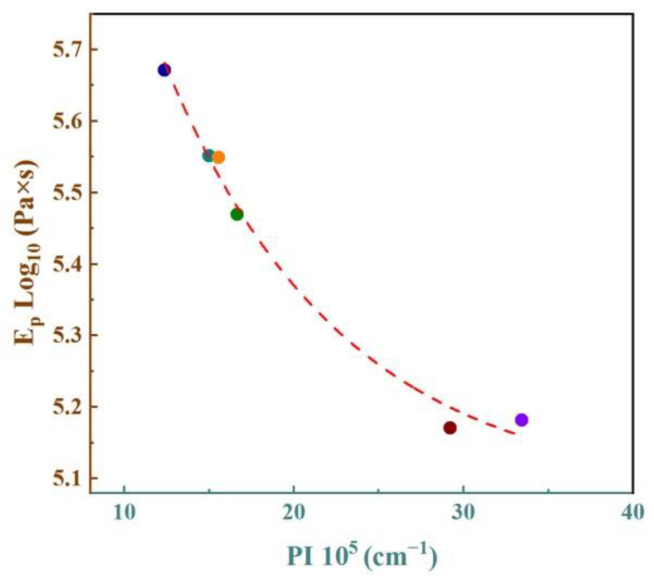
The fitting curve of PI and E_p_.

The PI and E_p_ of IC-00 to IC-50 samples are presented in Figure 8. With increasing the proportion of IC, PI decreased firstly and then increased, while E_p_ was the opposite. These variations indicate that the addition of IC reduces the pyroplastic deformation and increases the viscosity in industrial slag ceramics. Furthermore, controlling the additional proportion of IC prevents deformation and enhances the firing stability. The appropriate increasing proportion of IC resulted in a higher content of Fe-bearing diopside in the ceramic samples (Table 4). Fe-bearing diopside might act as a “skeleton” to support ceramic samples and to reduce their deformation due to stress, similar to the function of mullite in stoneware tiles and porcelain tiles [9,38,57]. However, the Fe_2_O_3_ and R_2_O components in IC affected the cell structure of the Fe-bearing diopside, further destabilizing the “skeleton” structure. Therefore, an excessive proportion of IC led to an enlarged distortion of the Fe-bearing diopside cells (Table 5), a reduction in the structural stability of the “skeleton” and an increase in the pyroplastic deformation of the ceramic samples. In addition, increasing the content of R_2_O in the formulation decreased the production temperature of the liquid phase and accelerated its viscous flow in the ceramic samples. When the liquid phase covered the grains’ surface or filled their gaps and significantly reduced the apparent porosity and water absorption close to zero, the deformation of the ceramic samples might increase as a result [33]. Accordingly, we speculated that the effect of IC on the pyroplastic deformation and viscosity of industrial slag ceramics was indirect and complex. A clear correlation between the PI and E_p_ of the ceramic samples is shown in Figure 9. The equation describing this relationship is y = 1.9565 × exp(−x/10.5172) + 5.078 with an R^2^ = 0.9896 and an adj. R-square = 0.9827. Obviously, pyroplastic deformation and viscosity are closely correlated, and a larger deformation follows a lower viscosity for industrial slag ceramics. This result is consistent with the analysis of the relationship between pyroplastic deformation and liquid phase viscosity in vitreous ceramics by Deng et al. [34].

The IC-30 sample had the smallest PI (12.37) and the largest E_p_ (log_10_5.67), which means it has a high ability to resist deformation and good high-temperature stability. At the same time, the IC-30 sample had the highest Fe-bearing diopside content, the densest microstructure, and the best physical properties. Consequently, the optimal IC amount was determined to be 30%.

Table 6 compares this work with other studies and the Chinese standard for ceramic tiles (GB/T 4100-2015). The physical properties of the ceramic samples obtained from this work are generally excellent, and the PI values are relatively low compared to other studies. Stoneware porcelain tiles are the main products manufactured in the current ceramic industry for floor and wall coverings. Compared with the standard for stoneware porcelain tiles, the samples’ water absorption and bending strength meet the requirements. The industrial slag ceramics of this work can be used as a substitute for stoneware porcelain tiles as floor and wall tiles.

## 4. Conclusions

In this study, high-performance industrial slag ceramics were prepared from Ti-extraction blast furnace slag and illitic clay. The effects of illitic clay on ceramic samples were summarized to four parts. (1) Phase: The main crystalline phase changed from akermanite to Fe-bearing diopside with increasing the proportion of illite clay in the ceramic samples. The content of Fe-bearing diopside increased firstly and then decreased (31.2% → 78.3% → 50.5%), while its cell volume increased continuously (440.6 Ǻ → 443.4 Ǻ). A positive linear correlation was found between the amorphous phase content and the proportion of illite clay. (2) Microstructure: The relatively dense microstructure of the ceramic samples was composed of tightly aggregated short columnar and granular grains and a few isolated pores; the mean value of the pore size was 0.26 μm, and the maximum value was 1.28 μm. Plate-like grains would disrupt the dense microstructure, causing the mean and maximum pore size values to increase to 0.39 μm and 3.2 μm, respectively. (3) Pyroplastic deformation and viscosity: Increasing the proportion of illitic clay led to a decrease and then an increase in the pyroplastic deformation of the ceramic samples, but the viscosity was the opposite; a larger deformation was associated with a lower viscosity. The appropriate addition of illitic clay contributed to reducing the pyroplastic deformation and enhancing the high-temperature thermal stability of the industrial slag ceramics. (4) Properties: The content of Fe-bearing diopside determines the bulk density and bending strength. The aggregation degree of grains and the amorphous phase content are the keys to reducing water absorption and apparent porosity. IC-30 sample had the highest Fe-bearing diopside content, 78.3%, relatively dense microstructure, and excellent physical properties, such as bulk density of 2.82 g/cm^3^, water absorption of 0.21%, apparent porosity of 0.6%, and bending strength of 62.17 MPa. It also had the lowest PI (12.37) and the highest viscosity (log_10_5.67). As a result, the optimum proportion of illitic clay was 30%.

## Figures and Tables

**Table 1:** Chemical compositions of raw materials (wt. %).

Sample	CaO	SiO_2_	Al_2_O_3_	TiO_2_	MgO	Fe_2_O_3_	Cl	R_2_O	Others	LOI
EBFS	32.44	19.23	11.03	8.09	6.87	2.24	8.96	0.84	2.06	8.24
IC	10.52	48.13	15.84	0.82	2.18	7.42	0.03	3.72	0.54	10.8
REBFS	31.63	25.43	14.44	10.41	8.18	2.96	2.70	0.50	1.63	2.12

**Table 2:** Ceramic formulas and proportions of raw materials (wt. %).

Formula	REBFS	IC
1	100	0
2	90	10
3	80	20
4	70	30
5	60	40
6	50	50

**Table 3:** Refinement factors and R/E values of the ceramic samples.

Samples	R-Factors	E-Factors	R/E
IC-00	0.1302	0.0883	1.47
IC-10	0.0982	0.0904	1.09
IC-20	0.0889	0.0832	1.07
IC-30	0.1080	0.0890	1.21
IC-40	0.0834	0.0850	0.98
IC-50	0.0746	0.0824	0.91

**Table 4:** Relative contents of phases in the ceramic samples.

Phases	Samples					
	IC-00	IC-10	IC-20	IC-30	IC-40	IC-50
Akermanite	43.8(3.4)	28.4(2.7)	12.8(1.8)	--	--	--
Fe-bearing diopside	31.2(2.7)	52.3(4.0)	73.4(5.2)	78.3(6.1)	65.0(5.0)	50.5(3.9)
Perovskite	14.4(1.1)	10.5(1.1)	6.1(0.8)	4.4(1.1)	1.2(0.5)	--
Spinel	5.3(1.0)	2.7(0.8)	1.0(0.5)	--	--	--
Anorthite	--	--	--	9.1(1.6)	20.5(2.4)	35.6(3.1)
Titanite	--	--	--	0.5(0.4)	4.6(1.1)	4.7(0.9)
Amorphous	5.3	6.1	6.7	7.7	8.7	9.2

**Table 5:** Cell parameters and volumes of Fe-bearing diopside.

Samples	a (Å)	b (Å)	c (Å)	V (Å^3^)	α (°)	β (°)	γ (°)
IC-00	9.743	8.849	5.319	440.6	90.0	106.082	90.0
IC-10	9.751	8.846	5.322	441.1	90.0	106.096	90.0
IC-20	9.751	8.846	5.322	441.1	90.0	106.109	90.0
IC-30	9.762	8.856	5.325	442.3	90.0	106.098	90.0
IC-40	9.768	8.876	5.320	443.2	90.0	106.086	90.0
IC-50	9.770	8.885	5.316	443.4	90.0	106.079	90.0

**Table 6:** Comparison of this work with ceramic samples derived from other studies and standard.

Raw Materials	Firing Temperature (°C)	Main Phase	Water Absorption (%)	Bending Strength (MPa)	Pyroplastic Deformation Index	Ref.
Steel slag + Kaolinite	1200	Quartz + Anorthite + Wollastonite	5.61~17.12	2~58	*	[58]
Steel slag + Base body	1140	Quartz + Anorthite + Diopside	0~15	<117	*	[11]
Blast furnace slag + Kaolin + Quartz	1210	Quartz + Anorthite + Pyroxene	0~3	39~49	*	[59]
Blast furnace slag + Clay + Kaolin + Limestone + Sand	1136	Quartz + Anorthite	11.73~18.2	*	*	[12]
Fly ash + Feldspar + Quartz	1300	Quartz + Mullite	*	*	~17	[60]
Clay + Feldspar + Quartz	*	*	0.5~3	>35	*	GB/T 4100-2015
Ti-extraction blast furnace slag + Illitic clay	1170	Fe-bearing diopside + Anorthite	0.21	62.17	12.37	This work

* is not provided with relevant information.

## Data Availability

Not applicable.
